# Cytogenetic Studies of 608 Couples with Recurrent Spontaneous Abortions in Northeastern Iran

**DOI:** 10.30699/IJP.2021.521514.2554

**Published:** 2021-07-06

**Authors:** Narjes Soltani, Farzaneh Mirzaei, Hossein Ayatollahi

**Affiliations:** 1 *Department of Hematology and Blood Bank, Faculty of Medicine, Cancer Molecular Pathology Research Center, Ghaem Medical Center Mashhad University of Medical Sciences, Mashhad, Iran*; 2 *Medical Genetic Research Center, School of Medicine, Mashhad University of Medical Sciences, Mashhad, Iran*

**Keywords:** Chromosomal Abnormalities, Inversions, Reciprocal Translocations, Recurrent Spontaneous Abortion (RSA), Robertsonian Translocations

## Abstract

**Background & Objective:**

One of the major genetic causes of recurrent spontaneous abortions is parental chromosomal abnormalities. The objectives of the study were to determine, compare and analyze the incidence and distribution of chromosomal abnormalities in couples with recurrent miscarriages from Northeastern Iran.

**Methods:**

This study was conducted at Ghaem Hospital, Mashhad, Iran. We evaluated karyotype results of 608 couples with history of recurrent spontaneous abortion. The standard method was used for culturing peripheral venous blood lymphocytes.

**Results:**

Chromosome aberrations were detected in 43 patients (3.54%), including 25 females and 18 males. Structural chromosomal abnormality was detected in 40 cases, including balanced translocations (25 cases), robertsonian translocations (4 cases), inversions (10 cases) and numerical chromosome aberrations (3 cases). Polymorphic variants were observed in 22 individuals.

**Conclusion:**

The frequency of chromosomal abnormalities in couples with Recurrent Spontaneous Abortion (RSA) in our study is 3.54%. Reciprocal translocation, pericentric inversions, robertsonian translocations, and numerical abnormality observed among couples who had experienced recurrent spontaneous abortions and that these couples might benefit from cytogenetic analysis.

## Introduction

Recurrent spontaneous abortion (RSA) is the loss of three or more consecutive pregnancies before 20-28 weeks of gestation; it is a prevalent clinical problem, which affects 1% to 2% of women. Some researchers believe that even two spontaneous miscarriages constitute recurrent abortions and deserve evaluation (1-3).

Approximately, 15-20% of all clinically detected pregnancies in women result in spontaneous abortions. The American Society of Reproductive Medicine recommends a thorough evaluation after three or more losses. RSAs are often related to factors such as: age of parents, genetic malformation, endocrine and autoimmune disorders, infectious diseases, environmental toxins and congenital or structural uterine abnormalities (4). 

Paternal and maternal chromosomal abnormalities play a significant role in early human age abnormal growth. The prevalence of chromosomal disorders in people with RSA varies between 2-8%. Approximately, 60% of spontaneous abortions in the first trimester of pregnancy have an abnormal karyotype. In spite of the fact that the most common chromosomal disorders in couples with RSA vary among populations, the frequency has been detected about 0.3-0.4% in the general population (5, 6).

Various cytogenetic studies have been performed to determine the type of chromosomal abnormalities in couples with RSA in several countries and different regions. Thus, cytogenetic investigation of parents with RSAs is essential in detection. Abnormalities such as reciprocal translocations, robertsonian translocations, and pericentric inversion are all associated with RSAs.

Chromosomal structural abnormalities in parents are main reasons behind recurrent miscarriage, because unequal crossing over during meiosis can result in chromosomal rearrangement of gametes with unbalanced chromosomal aberrations such as duplications or deletions. The clinical consequences of such unbalanced rearrangements are generally lethal for the embryo; it can lead to RSAs or giving birth to a malformed child (7).

The present study aimed to evaluate the frequency and types of chromosomal abnormalities in couples with RSA history in Northeast of Iran. The cytogenetic results can provide important information on genetic counselling and future genetic preventions; this study increases the knowledge of gynecologists and physicians about the prevalence and chromosomal anomalies that lead to repeated miscarriages (RM). It can also be used to compare our findings with previous reports and generate baseline data regarding chromosomal aberrations in the context of RM in this region.

## Material and Methods

The study, conducted between December 2010 and July 2019, included 608 couples with a history of two to 13 miscarriages, who referred to the Molecular and Cytogenetic Pathology Laboratory, Ghaem Hospital, Mashhad, Iran. An informed consent was taken from each patient as per the norms of the Institutional Ethics Committee. The history of the patients was recorded in a predesigned standard template to study the heredity pattern. All the referred couples were rigorously examined, and detailed clinical and obstetric histories were noted in prepared forms. We investigated couples’ basic information such as number of RSA, age, and other causes for abortion such as hormonal disorders, uterine malformations, and previously induced abortion(s). For routine cytogenetic analysis, peripheral venous blood samples were collected in heparinized vacutainers from each patient. For each case, two flasks of whole blood cells (0.5 mL) were cultured in 5 ml RPMI 1640 medium (Gibco, USA), containing 20% fetal bovine serum (FBS, Gibco, USA), an antibiotic mixture (10,000 units of Penicillin and 10 mg of stabilized streptomycin solution; SIGMA) and 10 μg/mL phytohaemagglutinin (Gibco, USA) at 37°C for 72 hours. A high-resolution technique was applied to peripheral blood cells by synchronization. The cells were incubated for another 4 to 5 hours after washing with thymidine inhibitor, then harvested in prometaphase with colcemid. Cultured cells were treated with 0.1 µg/mL of colcemid (Gibco-Invitrogen-USA) and then metaphase chromosomes were spread and stained using standard Giemsa–trypsin banding technique (8). In each case, 15 metaphase spreads were analyzed with Video Test-Karyo software Version 3.1, and when mosaicism was suspected, at least 50 metaphases were examined. Karyotype reports were based on the International System for Human Cytogenetic Nomenclature recommendations (ISCN) 2016.

## Results

**Fig 1:**
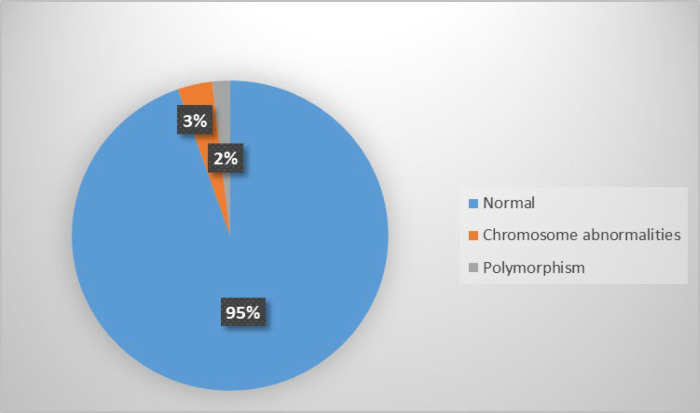
Pie chart for the percentage of abnormal karyotypes and polymorphisms observed in this study

**Fig 2:**
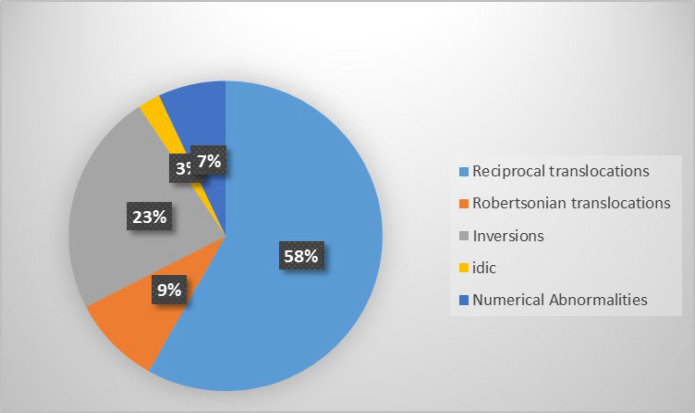
Pie chart for the percentage of different abnormalities observed in this study

In this study, Couples’ ages ranged from 19 to 64 years, with a mean of 30.41 years (SD=5.9) and the number of miscarriages ranged from two to 13. The 608 couples (1216 individuals) studied that according to the number of previous spontaneous abortion were showed in Table 1. Approximately, half of the patients had two miscarriages (49.7%) (Table 1). The mean number of miscarriages was 2.7 per couple (SD=1.07). Chromosome abnormalities were detected in 43 patients (3.54%), including 25 women and 18 men. Abnormal karyotypes are shown in Tables 2 and 3. Among 43 cases, 40 (93%) showed structural aberrations and three (7%) showed numerical abnormalities. Reciprocal translocations were identified in 25 cases, including 16 women and 9 men. Robertsonian translocations were seen in one man and three women. Inversions were identified in seven men and three women. In addition, there were 22 (1.8%) cases with three types of polymorphic variants including constitutional fragility of chromosome 16, pericentric inversion of chromosomes 1 and 9, enlarged heterochromatin in chromosomes 1, 16 and Y and extended satellites in chromosomes 14, 15 and 22 (Table 4). The pie charts for these results are shown in Figures 1 and 2.

**Table 1:** Couples grouped according to the number of miscarriages

Number of miscarriages	No .of couples	Percentage
2	302	49.7
3	214	35.2
4	50	8.2
5	21	3.5
6	10	1.6
7	3	0.5
8	4	0.6
9	3	0.5
13	1	0.2
Total	608	100

**Table 2:** Cytogenetic findings, number of miscarriages and maternal/paternal age in recurrent miscarriage cases with structural aberrations

Karyotypes	No .of miscarriages	Sex	Maternal/ Paternal age (years)
*Reciprocal translocation*			
6,XY,t(4;7)(q31;p22)	2	M	30
46,XX,t(14;21)(q24;q22)	4	F	30
46,XX,t(11;22)(q25;q11)	2	F	32
46,XY,t(4;5)(q34;q12)	2	M	28
46,XX,t(13;21)(q10;q10)	2	F	36
46,XX,t(11;22)(q22;q12)	2	F	25
46,XY,t(20;21)(q13.1;q22.2)	3	M	40
46,XY,t(4;7)(q31;q22)	2	M	33
46,XX,t(2;12)(q37.3;q24.1)	2	F	20
46,XY,t(4;7)(q31;q22)	2	M	31
46,XX,t(13;21)(q10;q21)	2	F	28
46,XX,t(6;10)(q22.1;q25.1)	2	F	43
46,XX,t(16;17)(p13.2;q25)	3	F	23
46,XX,t(1;2)(p36.2;q16)	4	F	41
46,XX,t(15;21)(q10;q10)	2	F	37
46,XX,t(11;22)(q24;q13)	4	F	34
46,XX,t(11;16)(q23;q24)	2	F	28
46,XX,t(6;7)(q25;p22)	3	F	33
46,XY,t(1;18)(p13;q21.3)	3	M	32
46,XY,t(1;16)(q21;q12.1)	2	M	35
46,XY,t(1;2)(q32;q13)	2	M	28
46,XX,t(7;15)(q31.2;q26.1)	3	F	26
46,XY,t(13;21)(q14;q22)	3	M	26
46,XX,t(13;21)(q31;q13)	4	F	25
46,XX,t(3;9)(p26;q31)	3	F	19
45,XX,idic(22)(p11.2)	2	F	31
*Robertsonian translocations*			
45,XX,rob(21;21)(q10;q10)	2	F	31
45,XY,rob(13;14)(q10;q10)	2	M	28
45,XX,rob(13;14)(q10;q10)	2	F	20
45,XX,rob(13;14)(q10;q10)	2	F	30
*Pericentric inversions*			
46,XY,inv(3)(p11q12)	5	M	32
46,XX,inv(5)(p15q15)	2	F	36
46,XY,inv(10)(p11.2q26)	2	M	28
46,XY,inv(10)(p11.2q26)	2	M	25
46,XY,inv(10)(p14q11.2)	2	M	25
46,XX,inv(12)(p11.2q11)	2	F	21
46,XY,inv(12)(p11.2q11)	2	M	30
46,XY,inv(18)(p11q11)	2	M	36
46,X,inv(X)(p22.3q26)	2	F	31
46,X,inv(Y)(p11q11)	2	M	32

**Table 3:** Cytogenetic findings, number of miscarriages and maternal/paternal age in recurrent miscarriage cases with numerical chromosomal abnormalities

*Numerical*	No .of miscarriages	Sex	Maternal/ Paternal age (years)
45,X[40]/46,XX[10]	2	F	36
47,XX,+mar[23]/46XX[27]	2	F	24
47,XY,+mar	5	M	30

**Table 4:** Polymorphic chromosomal variants in recurrent miscarriage cases

Polymorphic variants	No .of case	%
Pericentric inversion (9)(p11q13)	8	
fragility (16)(q23)	1	
15pstk+	1	
21pstk+	1	
14ps+	2	
15ps+	3	
22ps+	2	
1qh+	2	
1qh+,Yqh+	1	
6ph+	1	

## Discussion

In our study chromosome abnormalities were detected in 43 out of 1216 patients (3.54%) including 25 women and 18 men. Pursuant to literature, the prevalence of chromosomal abnormalities among couples who have recurrent miscarriages varies from zero to 21.4%. These differences may be related to sample size and inclusion and exclusion criteria (9).

Reciprocal translocations are one of the most frequent human chromosomal aberrations and occur in about 1 of 600 individuals in the general population (0.017%) (10), whereas they occurred at a frequency of about 2% (25 cases, including 16 women and 9 men) of couples in our study. Reciprocal translocations accounted for 58% of the aberrations observed in our study. Couples who have balanced reciprocal translocation have a 50% chance of RSA and a 20% risk of having children with an abnormal genetic constitution (11). “The mispairing of translocated chromosomes during the first meiotic division can give rise to different forms of segregation, resulting in aneuploidy of the translocated chromosomes in gametes” (12). 

Robertsonian translocation results from fusion of two acrocentric chromosomes on the centromic region (13). Its frequency is 0.1% in the general population (Link). Robertsonian translocations were seen in one man and three women (0.3%). Only chromosomes 13, 14, and 21 were found involved. The most frequent Robertsonian translocations were 13 and 14 translocations. Translocations involving both the homologues of chromosome 21 were observed in one woman. Translocation between chromosomes 13 and 14 was identified in two women and one man. Patients with Robertsonian translocations have only 45 chromosomes, but because deleted short arm regions do not have any essential genes, they yet have all necessary genetic material is present; the patients have normal phenotypes (14). However, they are at increased risk of unbalanced gamete and spontaneous abortion. Their gametes may either be normal, carry the fused chromosome, miss a chromosome, or have an extra acrocentric chromosome; the last two cases phenotypically affect the child or lead to abortion (15). In our study, 9% of observed aberrations had Robertsonian translocations.

Pericentric inversion was also associated with RSA. In a person with pericentric inversion, crossing over during meiotic division in their gametes may result in deletion or duplication of a segment in the involved chromosome (16). The mixture of monosomic and trisomic regions in a chromosome leads to miscarriage, unless the regions are small (17). Inversions were identified in seven men and three women and accounted for 23% of the aberrations observed in our study.

Numerical autosomal chromosomal aberration are not common among couples with repeated abortions, except for marker chromosomes. Sex chromosomal aneuploidy is the usual numerical chromosomal aberration in these patients (7). Two of the female subjects (0.16%) and one man in our study had a numerical abnormality. One woman was found to have mos 45,X[40]/46,XX[10] karyotype. A review articles showed that in the cases of mosaic Turner syndrome, the occurrence of spontaneous abortion ranged from 25% to 30% (18). The other woman showed mos 46,XX,+mar[23]/46XX[27] karyotype and the man had 47,XY,+mar karyotype pattern (Table 3). sSMC (small supernumerary marker chromosome) is an extra chromosome. sSMC can originate from any of the 24 different human chromosomes and its origin cannot be identified using conventional-banding cytogenetic techniques (19). An increased rate of recurrent abortions in sSMC carriers has been seen in 26-37% of the cases (20). This cannot precisely define the potential risk of spontaneous abortions caused by sSMCs (21). Infertile patients have a higher frequency of sSMCs than the general population (0.125% vs. 0.044%); it is also higher in infertile males (0.165%) than the females (0.022%) (18).

Chromosome variants or polymorphisms are microscopically detectable chromosome structures that vary in value, morphology, and staining pattern, and it is believed doesn't have any effect on the phenotype. Polymorphisms are inherited with a Mendelian pattern. Variable regions on the long arms of chromosomes 1, 9, 16, distal of the long arm of the Y chromosome, and the short arm of the acrocentric chromosomes (satellites) are the common polymorphisms of the human chromosomes (22). 

Pericentric inversion of chromosome 9, inv(9)(p11q12), is the most prevalent polymorphism in karyotypes. There is much debate about the effects of this polymorphism on phenotype, infertility, and recurrent miscarriages. It is commonly considered as a normal variant in the population without any phenotypic exposition. But in a lot of articles, it is believed that this polymorphism is a cause of infertility, recurrent abortions, congenital anomalies, and mental retardation. Its frequency is about 1.98% in the general population (23). The frequency of this polymorphism in our study was 0.65% (8 patients) (Table 4). By comparing these frequencies, it may be concluded that the observed inversion is not associated with recurrent miscarriage because its frequency is lower than its frequency in the general population.

In this study, the proportion of women with a chromosomal abnormality (58%) was higher than men (42%). In several previous studies, the number of female carriers with balanced chromosomal aberrations is reported more than male carriers in couples with recurrent abortions (23). “A proposed mechanism contributing to the higher incidence of female translocation carriers is that only one ovum matures each month, whereas male carriers release millions of sperm in every ejaculation, resulting in possible pre-zygotic selection against unbalanced gametes” (24). Another reason for more frequent female carriers than males is that structural chromosomal abnormalities in males may be the cause of severe oligozoospermia or azoospermia, and infertility. Chromosomal rearrangement could interrupt an important gene by position effects (25). Aneuploid sperm levels in fertile men are reported to be around 3-5% (25). In fact, all studies examining sperm aneuploidy levels in infertile men have shown a significant increase in aneuploid levels compared to fertile men (26).

## Conclusion

All obtained results confirm the importance of cytogenetic analyses in couples with recurrent abortions, because the cytogenetic results could provide important information for their genetic counseling and future genetic prevention. De novo rearrangements in the gametes of these carriers, cause birth defects risks in their offspring. Adequate genetic counseling strategies should also be offered, which could allow the couples to make an informed reproductive decision regarding subsequent pregnancies.
